# Maternal Immune Activation and Schizophrenia–Evidence for an Immune Priming Disorder

**DOI:** 10.3389/fpsyt.2021.585742

**Published:** 2021-02-17

**Authors:** Zahra Choudhury, Belinda Lennox

**Affiliations:** ^1^The Queens College, Medical Sciences Division, University of Oxford, Oxford, United Kingdom; ^2^Department of Psychiatry, University of Oxford, Oxford, United Kingdom

**Keywords:** schizophrenia, maternal, immune, complement, neurodevelopment

## Abstract

Schizophrenia is a complex neurodevelopmental disorder affecting around 19. 8 million people worldwide. The etiology of the disorder is due to many interacting genetic and environmental factors, with no one element causing the full spectrum of disease symptoms. Amongst these factors, maternal immune activation (MIA) acting during specific gestational timings has been implicated in increasing schizophrenia risk in offspring. Epidemiological studies have provided the rationale for this link with prevalence of maternal infection correlating to increased risk, but these studies have been unable to prove causality due to lack of control of confounding factors like genetic susceptibility and inability to identify specific cellular and molecular mechanisms. Animal models have proved significantly more useful in establishing the extent to which MIA can predispose an individual to schizophrenia, displaying how maternal infection alone can directly result in behavioral abnormalities in rodent offspring. Alongside information from genome wide association studies (GWAS), animal models have been able to identify the role of complement proteins, particularly C4, and display how alterations in this system can cause development of schizophrenia-associated neuropathology and behavior. This article will review the current literature in order to assess whether schizophrenia can, therefore, be viewed as an immune priming disorder.

## Introduction

Schizophrenia is a long-term mental health disorder with an etiology characterized by a range of positive symptoms (e.g., delusions and hallucinations), negative symptoms (e.g., loss of motivation) and cognitive deficits (e.g., working memory deficits) during early adulthood (1). Heritability is estimated to be 80% (2, 3), but susceptibility of developing schizophrenia is determined by complex interactions between genes and the environment (4, 5). Risk of developing the clinical phenotype is generally modeled on the “two-hit hypothesis” where the “first hit” is an early priming event disrupting neurodevelopment and establishing increased vulnerability to a “second hit” which tends to be environmental insults occurring later in life (6, 7). Genetic abnormalities are widely accepted as the “first hit,” but MIA may also serve this function (6).

Ecological studies were first to show the possible link between MIA via infection during pregnancy and increased schizophrenia risk in offspring. Following the 1957 influenza type A2 epidemic in Finland, maternal viral infection resulted in increased schizophrenia rates per 1,000 live births recorded in Helsinki (8). Other similar studies have attempted to replicate these findings, with some showing positive associations whilst others finding no association (9). Due to widely contradictory results, MIA animal models have been necessary to establish a causal relationship between MIA and schizophrenia-related abnormalities (10–13). These models have been pivotal in identifying molecular mechanisms that affect normal neurodevelopment in early life (10), most significantly changes to the complement system (4, 14). These changes cause neuronal connectivity and synaptic pruning dysfunction which may contribute to pathological characteristics seen in post-mortem schizophrenic brains (e.g., decreased cortical gray matter thickness), leading to social deficits observed in schizophrenic patients (14–16).

MIA falls under the category of prenatal maternal stress which broadly encompasses maternal infection/inflammation, obstetric complications, nutritional deficiencies and psychosocial stress. In particular, maternal psychological stress is documented to be a well-characterized early priming event resulting in both physiological and behavioral deficits in offspring, such as cardiovascular abnormalities, enhanced anxiety-like behavior and increased risk of schizophrenia and other psychotic disorders (17–19). Increased glucocorticoid secretion due to stimulation of the hypothalamic-pituitary-adrenal (HPA) axis may play a part, resulting in schizophrenia-like phenotypes, as seen with pregnant rats exposed to variable stress paradigms (20). Although maternal psychological stressors can have a large impact on schizophrenia risk in offspring, this essay will be focused on reviewing the evidence linking MIA in particular to schizophrenia in regard to epidemiological studies, MIA animal models and the complement system in order to evaluate whether schizophrenia can be classed as an immune priming disorder.

## Epidemiological Studies

Epidemiological studies have linked MIA to increased schizophrenia risk via infectious agents like influenza, herpes simplex virus 1 (HSV-1) and *Toxoplasma gondii (T. gondii)* (21–23). Ecological studies, primarily based on maternal influenza infection, identified this link, but there were many limitations associated with these. A notable limitation was diagnostic misclassification of influenza, with exposure in early studies being based upon maternal recall or whether the mother was in gestation during the epidemic, meaning around 70% of those in gestation during the 1957 influenza epidemic would have been misclassified as being infected whilst, in reality, being unexposed (8, 22, 24, 25).

A new approach was required to overcome this, leading to an increase in birth cohort studies using serological evidence, as summarized in Table 1. These studies used well-characterized birth cohorts and measured biomarkers in individual pregnancies using archived biological specimens obtained during pregnancy or early life of offspring (22). Results showed increased schizophrenia risk associated with maternal influenza infection in the first trimester (9), rather than the second trimester as found in ecological studies (8), and elevated levels of maternal antibodies against *T. gondii* (26, 27, 31, 32), as well as impaired cognitive functions displayed by HSV-1 seropositive offspring (23, 28, 29).

**Table 1:** Table reviewing serological evidence linking various infections to increased schizophrenia risk.

**Infection**	**References**	**Method**	**Population**	**Findings**	**Strengths**	**Limitations**
Influenza	Brown et al. (9)	Nested case-control study of the Prenatal Determinants of SZ birth cohort.	A subgroup of 12,094 offspring to mothers receiving obstetric care from the Kaiser Foundation Health Plan in Alameda County, California from 1959 to 1966.	Seven-fold increased SZ risk when exposed to influenza in 1st trimester. Three-fold increased SZ risk when exposed to influenza from mid-1st to mid-2nd trimester.	Prospective study in well-characterized birth cohort. Face-to-face psychiatric diagnostic assessment.	Serum samples frozen for 30+ years which may affect antibody levels. No inclusion of family history of SZ.
*T. gondii*	Brown et al. (26)	Nested case-control study of the Prenatal Determinants of SZ birth cohort.	A subgroup of 12,094 offspring to mothers receiving obstetric care from the Kaiser Foundation Health Plan in Alameda County, California from 1959 to 1966.	Two-fold increased SZ risk in offspring exposed to higher IgG antibodies against *T. gondii in utero*.	Prospective study in well-characterized birth cohort.	Serum samples frozen for 30+ years which may affect antibody levels. No inclusion of family history of SZ, maternal lifestyle or health. Modest group size.
	Mortensen et al. (27)	Cohort-based, case-control study in Denmark.	A group of 413 individuals registered in the Danish Psychiatric Case Register born from 1981 to 1999.	Increased SZ risk associated with increased IgG antibodies against *T. gondii* in offspring.	Large sample size. Included confounders like family history of SZ/mental health disorder.	Confined only to cases with early onset (18 years or younger), so cannot generalize to other age groups. 28% of SZ cases could not be located in the biobank which could have caused bias in results.
HSV-1	Yolken et al. (28)	Cognitive examination of SZ patients from the Clinical Antipsychotic Trials of Intervention Effectiveness (CATIE) sample.	A group of 1,308 individuals with SZ from 57 sites across all regions of the USA except Alaska.	Impaired visuomotor skills, verbal memory, attention and executive function in patients seropositive for HSV-1 only, not with other human herpesviruses.	Large sample size.	No inclusion of family history of SZ.
	Dickerson et al. (29)	Cognitive examination of SZ patients using the Repeatable Battery for the Assessment of Neuropsychological Status.	A group of 229 SZ patients from several treatment and rehabilitation programs in central Maryland aged 18 to 65 years old.	Impaired cognitive function in patients seropositive for HSV-1 only, not with other human herpesviruses.	Used solid-phase enzyme immunoassays which allow small volumes of serum samples to measure antibodies against a number of different antigens.	No evidence of CNS infection since did not measure levels of herpesvirus antibodies in the cerebrospinal fluid. Cross-sectional study so could not determine timing of initial HSV-1 infection or age of cognitive dysfunction development.
	Buka et al. (30)	Nested case-control study of three cohorts from the National Collaborative Perinatal Project (NCPP).	A group of 200 offspring with psychoses from a sample of pregnant women from Boston, Providence and Philadelphia.	1.8-fold increased SZ-associated psychosis risk in offspring of HSV-2 seropositive mothers. Elevated risk associated with offspring of mothers who did not use regular contraception and had frequent intercourse.	Prospective study in well-characterized birth cohort. Large sample size.	Losses to follow up may have caused bias. Overrepresentation of males and those from a high socioeconomic background in this sample.

*SZ, schizophrenia; Ig, immunoglobulin G*.

The mechanisms by which infection increases schizophrenia risk are only speculative and may either be infection-specific or work by a common causal mechanism (22). For example, the genome of *T. gondii* contains two genes encoding tyrosine hydroxylase which catalyzes L-DOPA production. L-DOPA is the precursor to dopamine, the dysregulation of which is well-established in schizophrenia pathology, thereby providing a potential infection-specific mechanism for increasing schizophrenia risk (33). However, there is currently limited evidence identifying *T. gondii* infection as a risk factor for schizophrenia, so larger birth cohort studies would be required to establish a more definitive association. Using human neurons derived from human induced pluripotent stem cells may also be useful in studying molecular mechanisms involved in *T. gondii* infection that lead to schizophrenia-associated pathogenesis. On the other hand, infections may act through common mechanisms by causing a pro-inflammatory state, increasing cytokine levels like IL-6 which has been shown to play a key role in behavioral abnormalities (22). A single injection of IL-6 during mid-pregnancy in rodents caused pre-pulse inhibition (PPI) and latent inhibition deficits in adult offspring, translating to sensorimotor gating deficits seen in schizophrenic patients, which can be prevented through administration of anti-IL-6 antibodies (34).

The prospective nature of serological studies has been powerful in establishing a link between MIA and increased schizophrenia risk through the use of qualitative measures of pathogenic markers in prenatal life. However, these studies are limited since they do not have the capacity to identify cellular and molecular mechanisms which affect neurodevelopment. Research in animal models provide a platform to overcome these limitations and potentially establish causality for these associations.

## MIA Animal Models

Initially, MIA was modeled using experimental mouse models of prenatal exposure to human influenza, where pregnant mice were infused with a mouse-adapted human influenza strain (35). Neuropathological signs were observed in the offspring, such as corticogenesis deficits, decreased hippocampal volume, decreased expression of γ-aminobutyric acid (GABA) markers, e.g., reelin, and behavioral abnormalities linked to schizophrenia (35). These results were extended to rhesus monkeys who showed decreased gray and white matter in cortical and parietal-cortical brain regions of neonates, verifying the relevance of rodent findings since corticogenesis in primates is more advanced and similar to humans (36). Viral MIA immune models were useful since they produced the full spectrum of immune responses caused by infections, but due to limitations associated with viral models such as the need for strict biosafety provisions and decreased control of immune response intensity and duration, animal MIA models moved onto using other immunogens such as polyinosinic:polycytidylic acid (polyI:C) to mimic viral infection, lipopolysaccharides to mimic bacterial infections and turpentine to mimic local inflammation (10). This article will particularly focus on polyI:C animal models due to the strength of their results, with Table 2 summarizing the main contributions these models have made to the study of schizophrenia pathophysiology.

**Table 2:** Main contributions made by polyI:C rodent models to the study of schizophrenia pathophysiology.

**References**	**Gestational day challenge**	**Main neurobiological findings**	**Main behavioral findings**
Meyer et al. (37)	9	Increases in IL-10 at 1 and 6-h post-challenge. Increases in IL-1β at 12 h post-challenge	Impairments in exploratory behavior, PPI, LI and spatial working memory. Enhanced locomotor response to systemic amphetamine.
Meyer et al. (38)	6, 9, 13 or 17	Increases in IL-10:TNF ratio in GD17 offspring.	GD6, 9 or 13 offspring display LI deficits.
Smith et al. (34)	12.5	-	Co-administration of IL-6 antibody with polyI:C prevents PPI, LI and exploratory/social deficits caused by polyI:C in offspring.
Meyer et al. (39)	9 or 17	GD9 offspring show decreased prefrontal D1 receptors in adulthood. GD17 offspring show decreased hippocampal NMDA receptor subunit expression. Potentiation of locomotor activity to amphetamine and decrease in reelin- and parvalbumin-expressing prefrontal neurons occur independently of polyI:C challenge timing.	GD9 offspring display impaired sensorimotor gating. GD17 offspring display impaired working memory and potentiation of locomotor activity to NMDA receptor antagonists.
Li et al. (40)	9 or 17	GD9 offspring display enlarged lateral ventricles in adulthood. GD17 offspring show 4th ventricle volume expansion.	GD9 offspring display PPI deficits.
Bitanihirwe et al. (41)	17	Sex-specific changes in neurotransmitter levels including: Reduced dopamine and glutamate in the PFC and hippocampus. Reduced GABA in hippocampus. Reduced glycine in the PFC.	Both male and female offspring display deficits in social interaction, anhedonic behavior, alterations in locomotor, and stereotyped behavioral responses to acute apomorphine treatment. Male offspring also display enhanced LI.
De Miranda et al. (42)	16	PolyI:C exposure inhibits embryonic neuronal stem cell replication and population of the superficial neocortex layers by neurons which is dependent on TLR3.	Impaired neonatal locomotor development and abnormal sensorimotor gating responses in adult offspring.
Abazyan et al. (43)	9	Prenatal polyI:C exposure in mhDISC1 mice causes decreased reactivity of the hypothalamic-pituitary-adrenal axis and attenuated serotonin neurotransmission in hippocampus. Reduced enlargement of lateral ventricles. Decreased volumes of amygdala and periaqueductal gray matter. Decreased density of dendritic spines of granule cells in hippocampus. Modulated secretion of cytokines in fetal brains. Altered levels of mhDISC1, endogenous mouse DISC1 and glycogen synthase kinase 3β.	Offspring display anxiety, depression-like phenotypes and altered social behavior. Behavioral effects only observed if mhDISC1 expressed throughout lifespan.
Coiro et al. (44)	12.5	Decreased number and turnover rates of dendritic spines at sites of excitatory synaptic inputs in cortex. Reorganization of pre-synaptic inputs with more spines contacted by both excitatory and inhibitory presynaptic terminals.	Increased repetitive behavior. Treatment with anti-inflammatory drug ibudilast prevents synaptic and behavioral impairments.
Duchatel et al. (45)	10 or 19	GD19 offspring showed increased NeuN+ IWMN density. Offspring exposed to polyI:C at both gestational stages showed increased SST+ IWMN density. 1st study to show MIA increases IWMN in adult offspring in a similar manner to what is seen post-mortem SZ brains.	-
Meehan et al. (12)	10 or 19	Male GD10 offspring show increased D1 receptor mRNA levels in nucleus accumbens.	GD19 offspring display transient working memory impairments. Male offspring exposed at either gestational timings display sensorimotor gating deficits.
Duchatel et al. (46)	10 or 19	Male GD19 offspring show significant increase in C4 gene expression in cingulate cortex.	-
Rahman et al. (11)	10 or 19	Decreases in SST mRNA in cingulate cortex and nucleus accumbens shell, and reduction of parvalbumin mRNA in infralimbic cortex regardless of polyI:C challenge timing. GD19 offspring display decreases in SSTR2 mRNA in cortex and striatum.	-

*IL, interleukin; LI, latent inhibition; TNF, tumor necrosis factor; TLR, toll-like receptor; mhDISC1, mutant human-disrupted in schizophrenia 1 gene; NeuN, neuronal nuclear antigen; IWMN, interstitial white matter neurons; SST, somatostatin; SSTR2, somatostatin receptor 2*.

In viral infection, double stranded RNA (dsRNA) is an intermediate in single stranded RNA replication or during symmetrical transcription of DNA viruses (10). Recognition of dsRNA by toll-like receptor 3 (TLR3) causes increased pro-inflammatory cytokine expression (10, 13). PolyI:C acts as a synthetic analog of viral dsRNA and mimics the acute phase response to viral infection (10, 13). The most significant finding from polyI:C rodent models would be how gestational timing of MIA exposure can influence the phenotype displayed by offspring (9, 11, 12). Pregnant mice were injected with polyI:C at either gestational day (GD) 9 or GD17 (39, 40, 47), translating to mid-first trimester and early second trimester, respectively, in humans (48). GD9 offspring were found to have phenotypes consistent with positive symptoms such as PPI deficits, latent inhibition deficits, decreased dopamine 1 (D1) and D2 receptor density in the prefrontal cortex (PFC) and increased tyrosine hydroxylase levels. Conversely, GD17 offspring showed phenotypes consistent with negative and cognitive symptoms such as N-methyl-D-aspartic acid (NMDA) receptor alterations, impaired reversal learning, working memory deficits, increased hyperlocomotion to MK-801 (an NMDA receptor antagonist) and decreased hippocampal expression of the N1 subunit of NMDA receptors (12). A limitation of this study is that these results were observed only in the C57 mouse strain. Therefore, further studies were undertaken in rats to provide evidence that these results were generalizable across species (12).

Pregnant Wistar rats were injected with polyI:C at GD10 or GD19 and their offspring undertook behavioral tests such as the acoustic startle response, delayed non-match to position testing and drug-induced locomotor activity assessments (12). Results from the rat study partially replicated findings from mouse studies; offspring exposed to late MIA showed working memory deficits and hyperlocomotion in response to MK-801, and offspring from both groups exhibited hyperlocomotion in response to amphetamine (12). Unlike the mouse study, the rat study showed decreased PPI in male offspring exposed to MIA at any gestational timing (12). This may be because the estrous cycle was not controlled for in female rats, meaning estrogen may have prevented the PPI disruption (12). On the contrary, a later study found female rats exposed to MIA in late gestation also exhibited sensorimotor gating deficits (11). These contradictory results suggest GD10 and GD19 may not be key sensitive periods of gestation for rats. Studies based on different gestational days with larger sample sizes should be undertaken to compare results.

Gestational timing of MIA is of great importance due to neuronal migration in neurodevelopment. A recent study reported decreased cortical and striatal, but not hippocampal, somatostatin (SST) mRNA in polyI:C adult rat offspring (11). This may be related to the distinct origins of these inhibitory neurons and the different timings involved in their migration (11). Cortical SST-containing inhibitory neurons derive from the medial ganglionic eminence (MGE), whilst 40% of hippocampal SST-containing inhibitory neurons derive from the caudal ganglionic eminence (CGE) (11). The MGE has an early dominant period of neurogenesis between GD9.5-GD13.5, whilst the CGE has a later period between GD12.5-16.5 (11). This explains why the hippocampus, and not the cortex, was spared when MIA was inflicted during GD10 and GD19, highlighting the great impact the specific timing of MIA can have on neuronal distribution (11). Further experiments should be conducted with MIA being inflicted during the key neurogenesis period of the hippocampus. If polyI:C offspring expressed characteristics associated with hippocampal dysfunction, such as memory and attention deficits, it would confirm whether MIA affects neurodevelopment through alterations in neuronal migration.

MIA animal model studies have also played a prominent role in identifying a link between complement system dysregulation and increased risk of schizophrenia-like phenotypes. The complement system is a key mediator of immunity, meaning that dysregulation of this system directly implicates the immune system in the etiology of schizophrenia. Complement proteins are involved in neurogenesis, neuronal migration and synaptic formation (4, 14), with C3 knockout mice showing synaptic elimination deficits (49) and impaired migration of neuroblasts (50). Early studies implicating complement system dysfunction in schizophrenia used hemolytic activity assays to measure overall complement component function in the blood (51–53). Results were varied, with some studies finding decreased total hemolytic activity in patients (52), whilst others found no differences between patients and controls (53). Complement system abnormalities are more reliably observed during infection; mothers of schizophrenic patients with increased fetal antibodies against adenovirus and HSV-2 also had higher C1q levels, indicating an early neurodevelopmental role for the complement cascade that may be due to MIA (54). Western blot analysis of C1q in different brain regions of polyI:C adult rodent offspring showed increased C1q expression in the PFC compared to controls (55). Although increased C1q likely contributes to behavioral abnormalities, this hypothesis has not specifically been assessed, meaning further behavioral tests are required to confirm this.

A recent GWAS of schizophrenia confirmed a robust genetic association with the major histocompatibility (MHC) locus, strongly implicating immune dysfunction as a potential pathological mechanism in schizophrenia (56). More specifically, through the use of digital droplet polymerase chain reactions and analysis of single nucleotide polymorphism (SNP) data, overexpression of C4 (originating from the MHC locus), particularly the *C4A* gene, has been linked with increased schizophrenia risk, with *C4A* mRNA being elevated in post-mortem brain tissue from schizophrenic patients (15). Immunohistochemistry on brain sections showed majority of C4-positive cells to be in the hippocampus, and co-immunostaining with pre-synaptic (vesicular glutamate transporters 1 and 2) and post-synaptic (post-synaptic density protein 95) markers showed much of the C4 was localized at synaptic puncta (15). Additionally, C4 knockout mice showed reduced synaptic pruning, suggesting C4 overexpression may contribute to decreased synapse numbers observed in schizophrenia (15). A limitation of this investigation is that C4 is encoded for by the *C4A* and *C4B* genes in humans, whilst encoded by a single *C4b* gene in mice, meaning these results may not directly translate to humans (15).

The involvement of C4 in synaptic pruning was rigorously studied by Comer et al. through C4 overexpression in mouse models (16). Results show dendritic spine abnormalities in C4-overexpressing mice which is consistent with post-mortem studies of schizophrenic patients (57). Changes in dendritic spines were most prominent in L2/3 of the PFC, but abnormalities have also been observed in other regions like the frontal lobe, temporal lobe and hippocampus (57). Therefore, targeting different areas with *in utero* electroporation (IUE) would be required to see if C4 overexpression decreased dendritic spine density in other areas also (16). Additionally, filopodia play a large role in synaptogenesis (58); decreases in the number of filopodia seen in these mice mean C4 may cause dysregulation of filopodia-dependent synapse formation in early development, resulting in abnormal synaptic elimination and circuitry that is seen in schizophrenia (16). Findings of decreased excitatory synaptic drive show how these structural abnormalities in dendritic spine density result in functional changes in connectivity (16). They also found excessive microglial engulfment of synaptic material was driven by C4 overexpression (16). Previous studies have showed that phagocytosis of synaptic material by microglia to be a necessary aspect of normal brain wiring (59), meaning the complement system may mediate brain circuitry, resulting in pathology when dysregulated. As well as genetic and molecular associations between C4 overexpression and schizophrenia, Comer et al. have found C4 overexpression in the frontal cortex to be sufficient in changing social interactions of both juvenile and adult mice, with results suggesting that overexpression of C4 can lead to long-term changes in PFC circuitry (16).

These results are the first to have shown a direct causal link between increased C4 levels and PFC dysfunction, and how this can lead to hypoconnectivity and abnormal synaptic functioning that drives expression of behavioral phenotypes seen in schizophrenia. Although it is recognized that schizophrenia is caused by a wide range of etiological factors, strong genetic, molecular and behavioral evidence has been provided by Comer et al. that altering the expression of only the *C4* gene is sufficient in mice to cause schizophrenia-like pathology. Future studies should focus on identifying whether changes in C4 expression in other brain areas can cause other schizophrenia-related phenotypes such as memory deficits.

Additionally, Comer et al. identified a critical developmental time period, around E16 in mice, during which PFC circuits are more vulnerable to changes in C4, highlighting the importance in the timing of gestational insults. This is further strengthened by a study using polyI:C MIA rodent models which, through qPCR, found a significant increase in *C4* gene expression in the cingulate cortex of offspring infected at GD19 only. This provides direct evidence, paired with results from Comer et al., that late MIA can cause schizophrenia-like deficits (46). Future studies could utilize transcriptomic and proteomic technology to delineate the molecular pathways underlying the relationship between late gestational MIA and C4 overexpression.

## Discussion

After review of epidemiological studies and animal models of MIA, it can be argued that schizophrenia can be regarded as an immune priming disorder, with MIA increasing the vulnerability of an individual to neuropathological effects of other environmental insults. Animal models have provided a wealth of information highlighting interactions between early neurodevelopmental disturbances and psychosis, showing strong face, construct and predictive validity (60). In particular, polyI:C models have been able to model behavioral, neuroanatomical and neurochemical changes seen in schizophrenic patients, including increased mesencephalic dopamine neuronal density and decreased NMDA receptor function in the PFC, consistent with the dopaminergic and glutamatergic hypotheses of schizophrenia (13). The obvious limitation of animal models is that many clinical manifestations of schizophrenia, e.g., hallucinations and delusions, are impossible to detect in animals (60). Therefore, behavioral, anatomical and physiological characteristics must be examined through translational models rather than modeling the whole syndrome (60). Although polyI:C models cannot mimic the full spectrum of immune responses, the relevance of this model in preclinical studies is not undermined since it does not influence its capacity to mimic schizophrenia-like behaviors and neuropathology (13, 60).

Animal models have provided strong evidence for the role of MIA in schizophrenia pathology, emphasizing the importance of gestational timing of infection in neurodevelopment and behavior. More specifically, MIA in late gestation causes increased C4 expression seen in schizophrenic patients. Subsequently, C4 overexpression has been proven to cause hypoconnectivity in the PFC and social deficits that are characteristic of schizophrenia, displaying how MIA is sufficient in causing schizophrenia-like phenotypes. Future studies utilizing modern functional imaging techniques are required to elucidate this relationship in humans and further confirm the contributing factor of C4 overexpression in schizophrenia development.

Although MIA may predispose individuals to schizophrenia, it is neither sufficient, nor necessary by itself for the development of the disease. Instead MIA can be viewed as a “priming” event, increasing vulnerability to the disorder, acting as a “first hit” toward the development of the disorder. The role of complement proteins in MIA is simply one aspect, among many, of immune dysfunction schizophrenia. Furthermore, the effect may also likely to be non-specific. Maternal stress is also a significant factor resulting in increased risk of different psychiatric disorders amongst offspring, potentially due to its effect on immune molecules like cytokines, with elevated maternal pro-inflammatory cytokines being associated with abnormal neurodevelopment (61–63). Taken together, a “multi-hit threshold model” encompassing different aspects of neurodevelopment would more accurately represent the dynamic interaction between genes and the environment in schizophrenia development (64).

Understanding the neuropathological origins of schizophrenia is important for detecting increased disease risk at an early stage and the development of preventative therapies. Detection of significant immune molecules, such as C4, may allow development of medication that could target these molecules or their receptors. Additionally, the identification of a key window of vulnerability provides a critical time period where such preventative therapies would be most efficacious. However, preventative therapies for neurodevelopmental disorders are controversial and would require extensive evaluation on both a scientific and ethical basis before being implemented in a clinical setting. Nevertheless, further investigation into the immune basis of schizophrenia could provide many potential therapeutic opportunities to improve treatment of this disorder.

## Author Contributions

ZC prepared the manuscript and constructed the accompanying tables. BL provided guidance with the structure of the manuscript and contributed to revision and proofreading. Both authors contributed to the article and approved the submitted version.

## Conflict of Interest

The authors declare that the research was conducted in the absence of any commercial or financial relationships that could be construed as a potential conflict of interest.
